# Integrated Analysis of Transcriptome and Metabolome Reveals Distinct Responses of *Pelteobagrus fulvidraco* against *Aeromonas veronii* Infection at Invaded and Recovering Stage

**DOI:** 10.3390/ijms231710121

**Published:** 2022-09-04

**Authors:** Xianhui Ning, Ye Peng, Peng Tang, Yiran Zhang, Lingling Wang, Wenwen Zhang, Kai Zhang, Jie Ji, Shaowu Yin

**Affiliations:** 1 College of Marine Science and Engineering, Jiangsu Province Engineering Research Center for Aquatic Animals Breeding and Green Efficient Aquacultural Technology, Nanjing Normal University, Nanjing 210023, China; 2 Co-Innovation Center for Marine Bio-Industry Technology of Jiangsu Province, Lianyungang 222005, China

**Keywords:** *Pelteobagrus fulvidraco*, transcriptome, metabolome, immune defense, distinct responses

## Abstract

Yellow catfish (*Pelteobagrus fulvidraco*) is an important aquaculture fish susceptible to *Aeromonas veronii* infection, which causes acute death resulting in huge economic losses. Understanding the molecular processes of host immune defense is indispensable to disease control. Here, we conducted the integrated and comparative analyses of the transcriptome and metabolome of yellow catfish in response to *A. veronii* infection at the invaded stage and recovering stage. The crosstalk between *A. veronii*-induced genes and metabolites uncovered the key biomarkers and pathways that strongest contribute to different response strategies used by yellow catfish at corresponding defense stages. We found that at the *A. veronii* invading stage, the immune defense was strengthened by synthesizing lipids with energy consumption to repair the skin defense line and accumulate lipid droplets promoting intracellular defense line; triggering an inflammatory response by elevating cytokine IL-6, IL-10 and IL-1β following PAMP-elicited mitochondrial signaling, which was enhanced by ROS produced by impaired mitochondria; and activating apoptosis by up-regulating caspase 3, 7 and 8 and Prostaglandin F1α, meanwhile down-regulating FoxO3 and BCL6. Apoptosis was further potentiated via oxidative stress caused by mitochondrial dysfunction and exceeding inflammatory response. Additionally, cell cycle arrest was observed. At the fish recovering stage, survival strategies including sugar catabolism with D-mannose decreasing; energy generation through the TCA cycle and Oxidative phosphorylation pathways; antioxidant protection by enhancing Glutathione (oxidized), Anserine, and α-ketoglutarate; cell proliferation by inducing Cyclin G2 and CDKN1B; and autophagy initiated by FoxO3, ATG8 and ATP6V1A were highlighted. This study provides a comprehensive picture of yellow catfish coping with *A. veronii* infection, which adds new insights for deciphering molecular mechanisms underlying fish immunity and developing stage-specific disease control techniques in aquaculture.

## 1. Introduction

Yellow catfish (*Pelteobagrus fulvidraco*) represents an economically important fish famous for its excellent meat quality and has been widely cultured in China [1,2]. However, the farming industry of yellow catfish has been severely threatened by the outbreaks of bacterial diseases, especially those caused by *Aeromonas* spp. [3]. Among the *Aeromonas* species, pathogenic *Aeromonas veronii* is of particular concern due to its virulence causing acute death [4]. The typical symptoms associated with *A. veronii* include ascites, body surface ulcers, and hemorrhagic septicemia. *A. veronii* ubiquitously exists in fresh and brackish water, infecting a variety of cultured fish, such as *channel catfish* (*Ictalurus punctatus*), gibel carp (*Carassius auratus gibelio*), and spotted sea bass (*Lateolabrax maculatus*) [5,6,7], as well as terrestrial animals and human [8].

Recent studies uncovered the immune regulatory role of cellular metabolism. Metabolomics is an efficient strategy to survey the changes in metabolic profile in a complex biological system under stress conditions, such as infection, which facilitates the identification of metabolic biomarkers [9]. In fish, metabolome has been extensively used to detect the immune response against pathogen challenges. For example, metabolic differences were explored between surviving and dying individuals of *Streptococcus iniae*-infected tilapia, which proved *N*-acetylglucosamine as the key metabolite [10]; the skin metabolome was examined in *Cryptocaryon irritans*-induced yellow drum [11]. In yellow catfish, vitamin D-3 and iron metabolism modulated by hepcidin were reported to contribute to the anti-*Edwardsiella ictaluri* and anti-*A. veronii* infection, respectively [12,13]. However, the systematic detection of metabolic changes in yellow catfish during infection has not been documented.

The integration of metabolomic and transcriptomic information enables a comprehensive overview of biological processes to examine the underlying mechanism, which cannot be observed with a single omics. Metabolic changes, especially those associated with energy metabolism and intermediate metabolites, are highly affected by gene expression changes, and vice versa [14]. Genes and metabolites are closely linked to fit the cellular demands of coping with stress conditions such as pathogen infections, the cross-talk of which drew great attention in the fish immune response against pathogens [15,16,17].

In the present study, the integrated and comparative analyses of the transcriptome and metabolome were performed to explore the global changes of genes and metabolites, identify key biomarkers and pathways, and characterize different defense strategies of yellow catfish in response to *A. veronii* infection at the invaded stage and recovering stage. Our findings provide valuable resources for future studies on immune mechanisms and stage-specific disease control of yellow catfish.

## 2. Results

### 2.1. Transcriptome Data Processing

**Figure A1:**
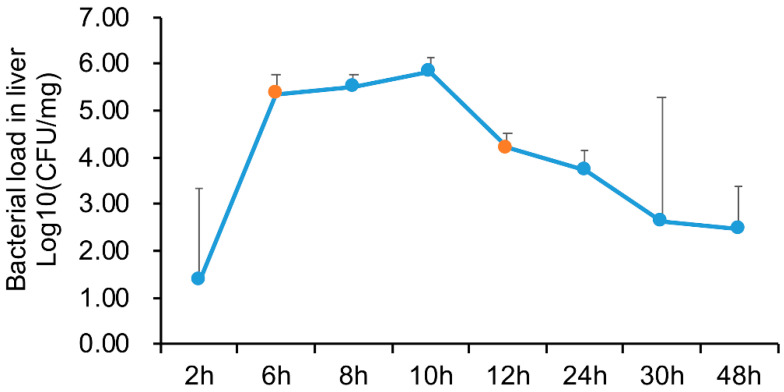
Bacterial load in liver of yellow catfish infected with *Aeromonas veronii*. yellow catfish was injected with *A. veronii*, and the bacterial load in liver was determined at 2, 6, 8, 10, 12, 24, 30 and 48 h. Data are shown as means ± SD.

According to the bacterial loads in the liver at different time points after *A. veronii* challenge, tissue dissemination of yellow catfish at 6 h and 12 h were among the increasing and declining trends in bacterial number (Figure A1), which were considered as invaded stage (IN) and recovering stage (RE), respectively. Yellow catfish of IN and RE were used to examine the defense responses at the transcriptional level. Nine libraries were constructed with RNA from livers in the control group (CT) and *A. veronii*-infected group (IN and RE), with biological triplicates in each group. The sequencing results showed that a total of 757,662,434 raw reads were obtained. After trimming and quality control, 755,522,536 (99.72%) reads passed the filter process and were termed high-quality (HQ) reads. The ribosomal RNAs were removed from these HQ reads, and the remaining HQ (RHQ) reads were mapped to the reference genome, which revealed a high mapping rate ranging from 93.37% to 95.32% (Table 1). In total, 23,807 annotation genes of yellow catfish were identified.

### 2.2. Identification of Differentially Expressed Genes (DEGs) at Invaded Stage and Recovering Stage

**Figure 1:**
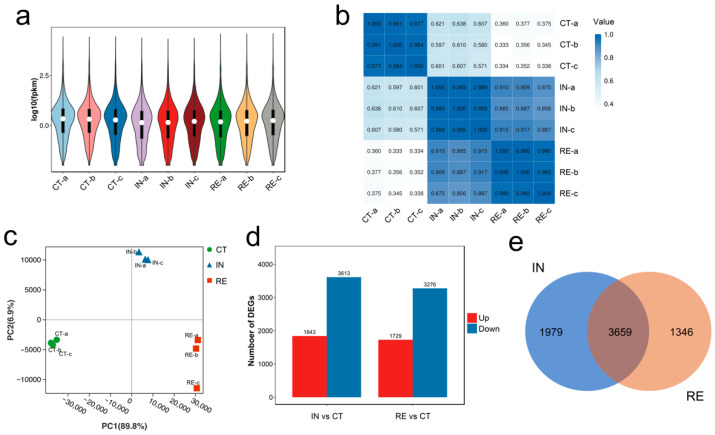
Expression features of all genes and characterization of differentially expressed genes (DEGs): (**a**) Violin plot showing the expression patterns of genes in biological triplicates of different groups. For convenience, “CT”, “IN”, and “RE” indicate the control group, *Aeromonas veronii*-infected groups at invaded stage and recovering stage. Within each group, “a”, “b” and “c” indicate biological triplicates; (**b**) The correlation matrix of gene expression among biological triplicates of different groups; (**c**) PCA plot exhibiting the variations of genes among groups with different treatments; (**d**) Number of DEGs in group IN and RE. “Up” and “Down” indicate up- and down-regulated expression, respectively; (**e**) Venn diagram exhibiting overlapping DEGs in group IN and RE.

The expression profiles of genes in groups CT, IN and RE were shown in the violin plot (Figure 1a). Within the biological triplicates of each group, the mRNA expression exhibited high repeatability with correlation coefficients > 0.97 (Figure 1b), reflecting the robustness of the sequencing data. The principal component analysis (PCA) was applied to recognize the sample relationship, which showed a clear separation among the three groups (Figure 1c). Specifically, the first principal component (PC1) explained 89.8% of the treatment variance, which discriminated the infected samples from the controls, indicating whether the fish were infected; PC2 explained 6.9% of the treatment variance, which discriminated samples at the invaded stage from that at recovering stage, indicating whether the fish were recovered from infection (Figure 1c).

The differential expression analysis was carried out based on the sample relationship, which showed that 5456 and 5005 genes were differentially expressed at the invaded stage and recovering stage, respectively, compared to that in the control group (Figure 1d). Specifically, 1843 and 3613 genes exhibited up- and down-regulated expression at the invaded stage, and 1729 and 3276 genes exhibited up- and down-regulated expression at recovering stage (Figure 1d). As shown in the Venn plot (Figure 1e), a total of 6802 DEGs were determined in *A. veronii*-infected yellow catfish, among which 3659 were differentially expressed continuously, 1797 were specifical DEGs at the invaded stage, and 1346 were specifical DEGs at recovering stage (Figure 1e, Table S1).

### 2.3. KEGG Functional Enrichment of DEGs

**Figure 2:**
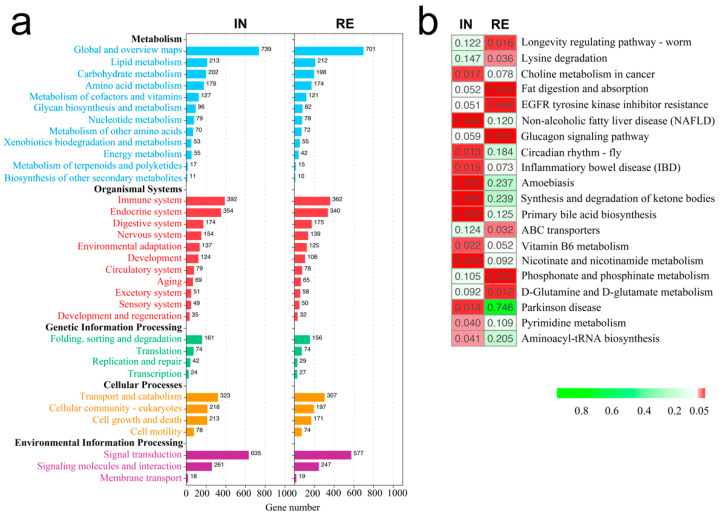
KEGG functional enrichment of DEGs in *Aeromonas veronii*-infected groups at invaded stage (IN) and recovering stage (RE): (**a**) Commonly enriched KEGG classes at level 2; (**b**) Exclusively enriched KEGG pathways by DEGs in IN or RE. The color bar indicates the *p* value of the enriched pathway. DEGs, differentially expressed genes.

To detect the biological processes engaged by DEGs in group IN and RE, KEGG functional analysis was performed accordingly. The result exhibited that 103 and 95 pathways were significantly enriched by DEGs at the invaded stage and recovering stage, respectively, which were summarized into five main categories in level two of KEGG (Figure 2a). In the category of metabolism, lipid metabolism, carbohydrate metabolism and amino acid metabolism were among the top involved pathways; in the category of organismal systems, immune system was the most involved pathway; in the category of cellular processes, transport and catabolism, cellular community, and cell growth and death were significantly enriched (Figure 2a). Except for the common categories, pathways associated with lipid synthesis (synthesis and degradation of ketone bodies, primary bile acid biosynthesis, and choline metabolism in cancer), and liver damage (non-alcoholic fatty liver disease [NAFLD], inflammatory bowel disease [IBD], and amoebiasis) were exclusively over-represented at invaded stage (Figure 2b), while pathways associated with nutrition degradation and transport (lysine degradation, fat digestion and absorption, and ABC transporters), D-Glutamine and D-glutamate metabolism, homeostasis (EGFR tyrosine kinase inhibitor resistance and glucagon signaling pathway) and longevity regulating were over-represented at recovering stage (Figure 2b).

### 2.4. Characterization of Metabolomic Profiles

**Figure 3:**
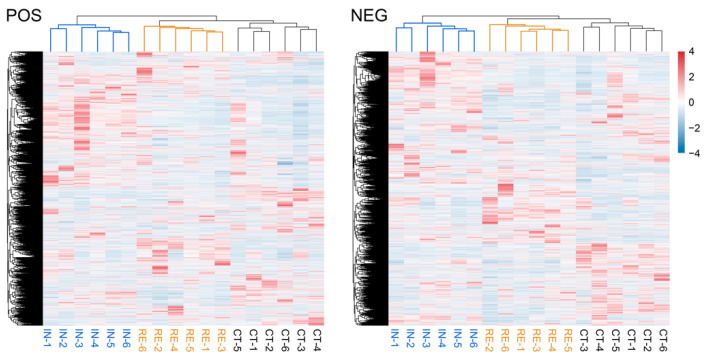
The hierarchical clustered relationship and the expression patterns of metabolites from the positive ion mode (POS) and negative ion mode (NEG) in different groups. For convenience, “CT”, “IN”, and “RE” indicate the control group, *Aeromonas veronii*-infected groups at invaded stage and recovering stage.

**Figure A2:**
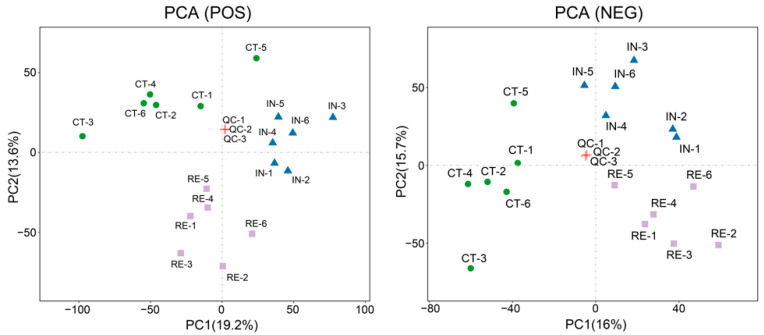
PCA plot exhibiting the result of quality control of metabolome data. The densely distributed of QC samples indicates the high reliability of the metabolome data.

To maximize the metabolite coverage and detection efficiency, both positive ion mode (POS) and negative ion mode (NEG) were applied in the metabolomic analysis. The result of quality control showed that the quality control (QC) samples were densely overlapped in the PCA analysis (Figure A2), indicating the high reliability of the datasets. Based on the qualitative and quantitative detections, a total of 8994 and 8345 metabolites were identified in the positive and negative modes, respectively. The hierarchical clustered relationship and the expression patterns of the metabolites from all samples in both POS and NEG modes were estimated (Figure 3). The result exhibited the clearly separated clusters among groups CT, IN and RE (Figure 3), indicating the discernible differences in the expression of metabolites among different treatments, which were suitable for subsequent analysis.

### 2.5. Identification of Differential Abundance Metabolites (DAMs) at Invaded Stage and Recovering Stage

**Figure 4:**
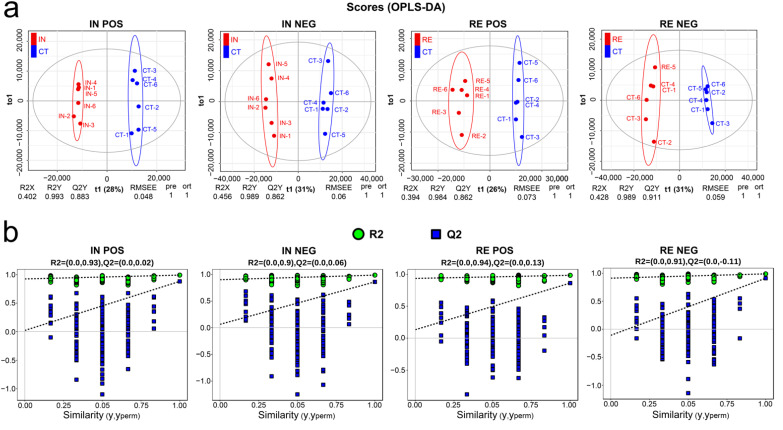
Multivariate statistical analysis for metabolites obtained from POS and NEG mode in group CT, IN and RE. For convenience, “CT”, “IN”, and “RE” indicate the control group, *Aeromonas veronii*-infected groups at invaded stage and recovering stage. (**a**) Orthogonal projection to latent structures-discriminant analysis (OPLS-DA) score plots. (**b**) Cross-validation and permutation tests.

To accurately capture the DAMs, multivariate statistical analysis was conducted using the OPLS-DA model, a method that could effectively reduce the complexity meanwhile enhancing the explanatory power contributing to maximizing the detection of differences between groups. The score plot of OPLS-DA showed that the metabolic profiles were clearly distinguished between group IN/RE and CT (Figure 4a). The Q2 value is a parameter indicating the predictive ability of OPLS-DA. For group IN and CT, the Q2 value in POS and NEG was 0.883 and 0.862, respectively (Figure 4a). For group RE and CT, the Q2 value in POS and NEG was 0.862 and 0.911, respectively (Figure 4a). The high values of Q2 indicate OPLS-DA as an excellent predictive model in this study. To be stringent, the OPLS-DA model was further verified by cross-validation and permutation test. As shown in Figure 4b, the low intercept values of Q2 (<0.20) certified the robustness and reliability of OPLS-DA. Therefore, OPLS-DA was effective to determine the metabolic differences in *A. veronii*-infected yellow catfish.

**Figure 5:**
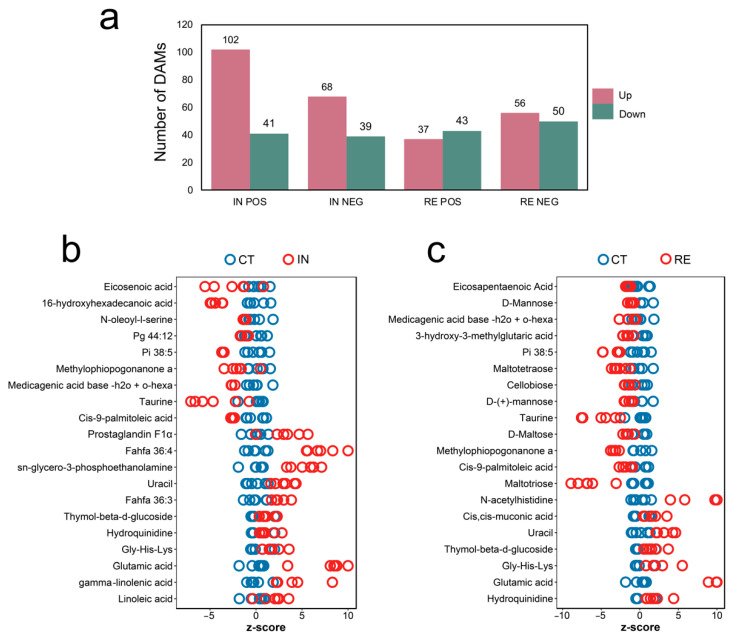
Differential abundance metabolites (DAMs) induced by *Aeromonas veronii* at invaded stage (IN) and recovering stage (RE). (**a**) Number of DAMs in group IN and RE. “Up” and “Down” indicate up- and down-regulated expressions. Z-score plots showing the top 20 DAMs in group IN (**b**) and group RE (**c**).

According to the variable importance in projection (VIP) score of OPLS-DA, the differential metabolites were identified with the threshold of VIP ≥ 1 and *p* < 0.05. As shown in Figure 5a, a total of 143 (102 up- and 41 down-regulated) and 107 (68 up- and 39 down-regulated) DAMs were obtained in group IN in POS and NEG, respectively (Table S2). In group RE, 80 (37 up- and 43 down-regulated) and 106 (56 up- and 50 down-regulated) DAMs were obtained in POS and NEG, respectively (Figure 5a, Table S2). Among the top 20 DAMs in group IN and RE, Glutamic acid, Uracil, Gly-His-Lys, and Thymol-beta-d-glucoside were commonly up-regulated, and Taurine were commonly down-regulated (Figure 5b,c); lipids such as FAHFA (fatty acyl esters of hydroxy fatty acid) family members Fahfa 36:3 and Fahfa 36:4, Gamma-linolenic acid and Linoleic acid, as well as Prostaglandin F1α, were specifically increased at invaded stage (Figure 5b), while carbohydrates such as D-Mannose, D-(+)-mannose, Maltotetraose, Maltotriose, and Cellobiose, were specifically decreased at recovering stage (Figure 5c). Except for the top 20 DAMs, glutamine was significantly up-regulated at both stages; lipids Glycerol 3-phosphate and Beta-glycerophosphate were found to be enhanced at the invaded stage, while antioxidants Glutathione (oxidized) and Anserine were exclusively enhanced at recovering stage (Table S2).

### 2.6. Functional Enrichment of DAMs

**Figure 6:**
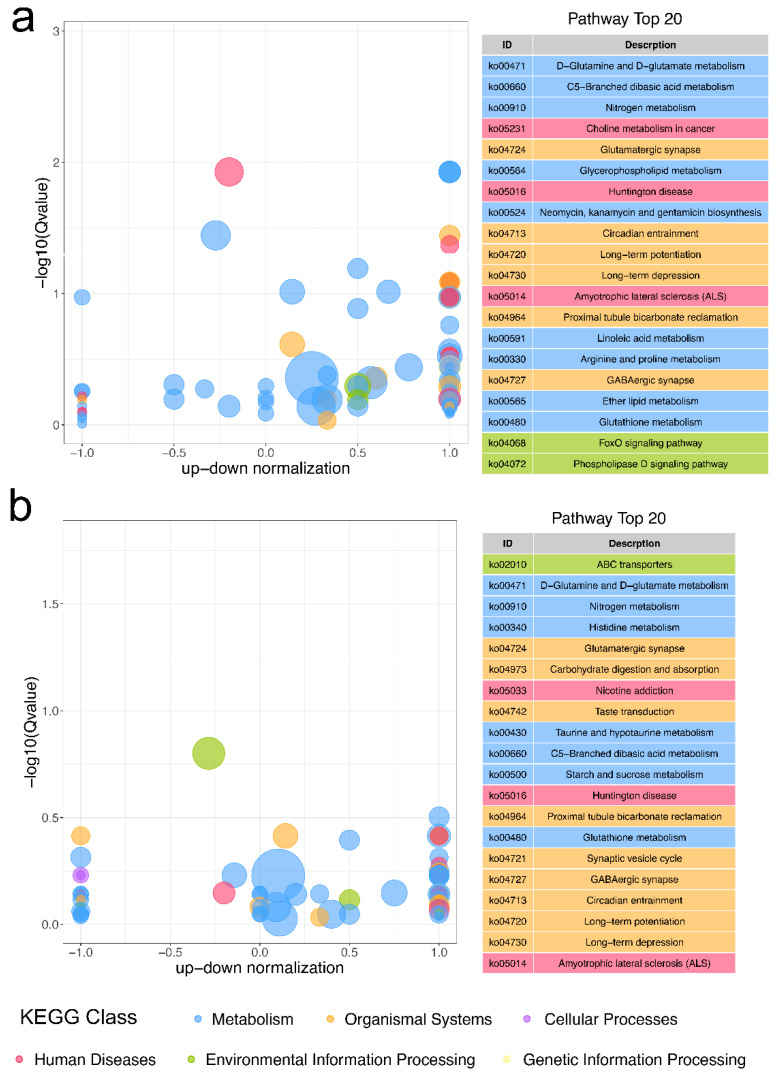
The top 20 most enriched KEGG pathways by DAMs in group IN (**a**) and group RE (**b**). The bubble size represents the number of DAMs. DAMs, differential abundance metabolites. For convenience, “IN” and “RE” indicate the *Aeromonas veronii*-infected groups at the invaded stage and recovering stage, respectively.

To determine the biological processes affected by DAMs, KEGG analysis was performed at the metabolic level. The top 20 remarkably enriched pathways for group IN and RE were commonly related to carbohydrate metabolism (C5-Branched dibasic acid), amino acid metabolism (D-glutamine and D-glutamate, and Glutathione), neuronal regulation (Glutamatergic and GABAergic synapse, and long-term potentiation and depression), and neurodegenerative disorder (Huntington disease and Amyotrophic lateral sclerosis) (Figure 6). At the invaded stage, immune-related pathways (neomycin, kanamycin and gentamicin biosynthesis and FoxO signaling), and lipid metabolism-related pathways (Glycerophospholipid, Linoleic acid, Ether lipid, Phospholipase D signaling, and Choline metabolism in cancer) were over-represented (Figure 6a). At the recovering stage, coinciding with pathways enriched by DEGs, nutrition degradation-related pathways (carbohydrate digestion and absorption, starch and sucrose metabolism, and ABC transporters) were over-represented (Figure 6b).

### 2.7. Integrated Analysis of Transcriptome and Metabolome in Response to A. veronii Infection

**Figure 7:**
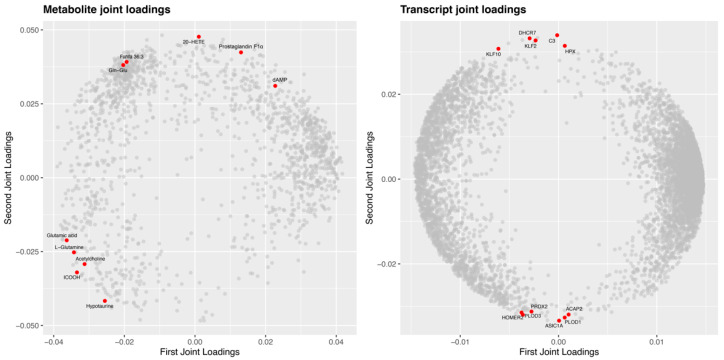
Joint loading plots of DAMs and DEGs. The plot was constructed based on O2PLS (two-way orthogonal PLS) model in the integrated analysis of transcriptome and metabolome of *Aeromonas veronii*-infected yellow catfish. In the plot, the farther a gene is located from the origin, the more closely it is related to the metabolites, and vice versa. DAMs and DEGs with high correlations are indicated in red nodes. DAMs, differential abundance metabolites. DEGs, differentially expressed genes.

To systematically dig into the intertwined information at transcriptomic and metabolomic levels, O2PLS and common-pathway models were both applied. We firstly performed the O2PLS analysis, of which the contribution assessment with high components (R2Xcorr = 0.837, R2Ycorr = 0.941) reflected O2PLS as a good model to explain the total variations between pairwise comparisons in the present study. According to loading values of O2PLS, the joint loading plots of metabolite and transcript were determined (Figure 7), which revealed the general correlation strength between genes and metabolites. In the plot, the farther the genes were located from the origin, the more closely they were related to the metabolites, and vice versa. As shown in Figure 7, metabolites such as Prostaglandin F1α, Fahfa 36:3, 20-HETE (20-hydroxyarachidonic acid), Gln-Glu, Glutamic acid and L-Glutamine, and ICOOH (Indole-3-carboxylic acid) were highly linked with genes. Moreover, among these metabolites, Prostaglandin F1α, Fahfa 36:3, and Glutamic acid were among the aforementioned top 20 DAMs. Genes associated with FoxO signaling pathway (Krueppel-like factor [KLF] 2 and Homer protein homolog 2 [HOMER2]), immunity (Complement C3 [C3] and Acid-sensing ion channel 1A [ASIC1A]), cell growth and death (KLF10 and ArfGAP with coiled-coil, ankyrin repeat and PH domains 2 [ACAP2]), antioxidant (Hemopexin [HPX] and Peroxiredoxin-2 [PRDX2]), cholesterol synthesis (7-dehydrocholesterol reductase [DHCR7]), and collagen synthesis (Procollagen-lysine,2-oxoglutarate 5-dioxygenase 1 [PLOD1] and PLOD3) were highly linked with metabolites (Figure 7).

**Figure 8:**
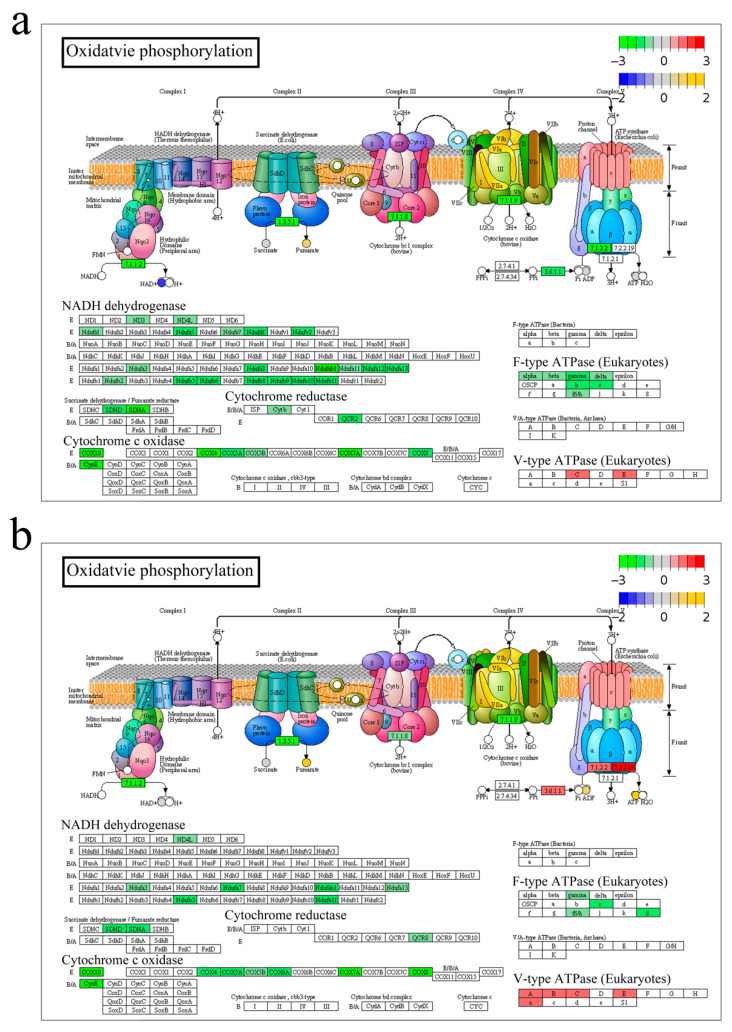
Oxidative phosphorylation pathway enriched by both genes and metabolites in group IN (**a**) and RE (**b**). Based on the pathway model in integrated analysis between transcriptome and metabolome of *Aeromonas veronii*-yellow catfish, oxidative phosphorylation pathway was enriched by both genes and metabolites at invaded stage and recovering stage. For convenience, “IN”, and “RE” indicate the *A. veronii*-infected groups at invaded stage and recovering stage, respectively.

**Figure 9:**
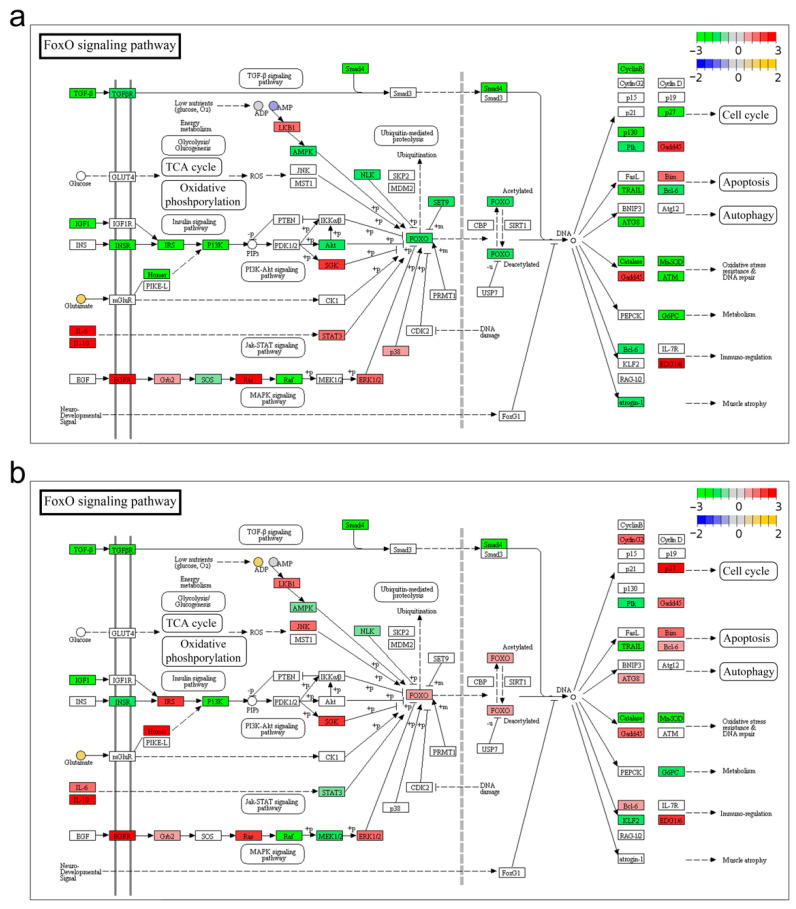
FoxO signaling pathway enriched by both genes and metabolites in group IN (**a**) and RE (**b**). Based on the pathway model in integrated analysis between transcriptome and metabolome of *Aeromonas veronii*-yellow catfish, FoxO signaling pathway was enriched by both genes and metabolites at invaded stage and recovering stage. For convenience, “IN”, and “RE” indicate the *A. veronii*-infected groups at invaded stage and recovering stage, respectively.

**Figure A3:**
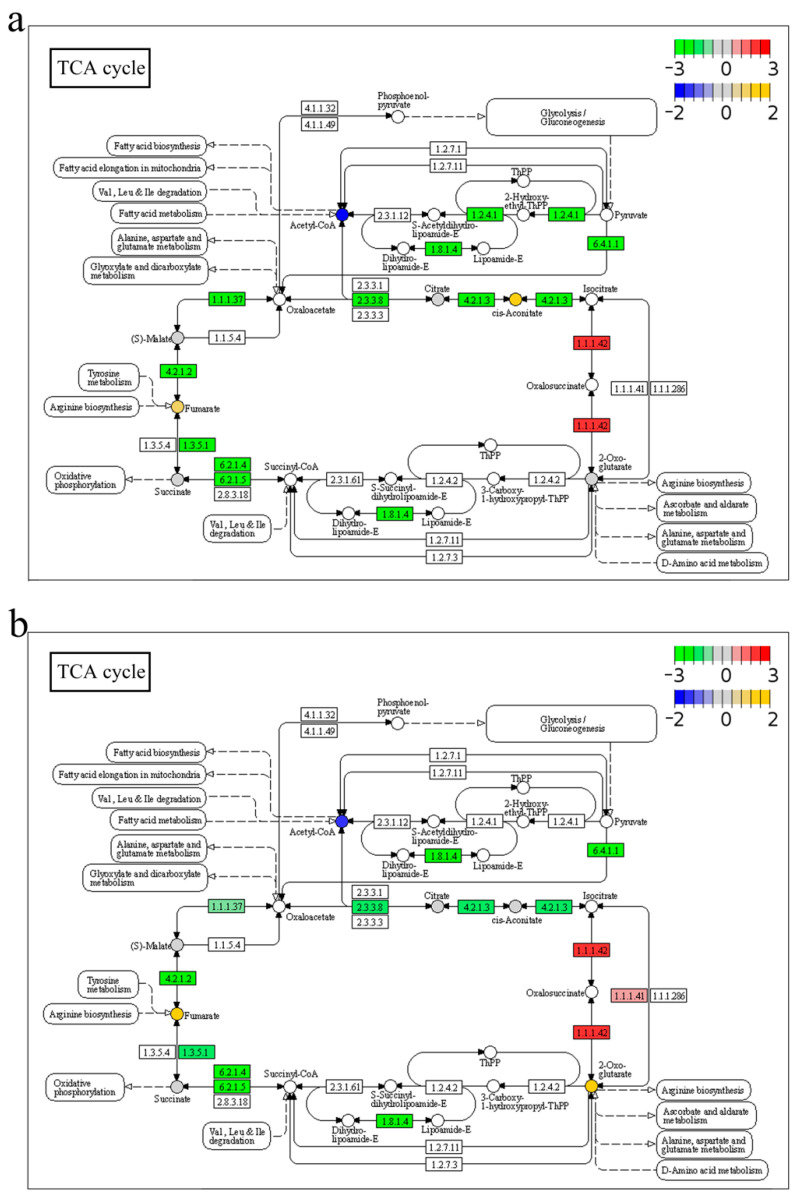
TCA cycle pathway enriched by both genes and metabolites in group IN (**a**) and RE (**b**). Based on the pathway model in integrated analysis between transcriptome and metabolome of *Aeromonas veronii*-yellow catfish, TCA cycle pathway was enriched by both genes and metabolites at invaded stage and recovering stage. For convenience, “IN”, and “RE” indicate the *A. veronii*-infected groups at invaded stage and recovering stage, respectively.

The pathway model was performed to uncover the biological activities coordinated and regulated by differential genes and metabolites. The results revealed that pathways associated with anabolism and catabolism (fructose and mannose metabolism, glycolysis, fatty acid biosynthesis, and glycerolipid metabolism), Energy synthesis (TCA cycle and oxidative phosphorylation), and immunity (FoxO signaling pathway, apoptosis, and autophagy) were mainly affected by *A. veronii* infection at both transcriptome and metabolism levels, which were graphically represented as TCA cycle (Figure A3), Oxidative phosphorylation (Figure 8), and FoxO signaling pathway (Figure 9).

In the TCA cycle (Figure A3), metabolite acetyl-CoA, and fumarate were down- and up-regulated, respectively, and most of the genes were decreased except the isocitrate dehydrogenase (EC1.1.1.41 and EC1.1.1.42) upon *A. veronii* stimulation. The amount of oxoglutaric acid (also known as α-ketoglutarate, AKG), and the expression of isocitrate dehydrogenase (including IDH2 and IDH3G) were significantly elevated in group RE. In Oxidative phosphorylation (Figure 8), most of the genes were depressed after infection. Metabolites NAD^+^ and AMP were down-regulated in group IN (Figure 8a and Figure 9a), while ADP and ATP were up-regulated in group RE (Figure 8b). The reduced expression of most of the members in NADH dehydrogenase, cytochrome c reductase and F-type ATPase in group IN (Figure 8a) were discernibly increased in group RE (Figure 8b), such as NADH-ubiquinone oxidoreductase 75 kDa subunit, mitochondrial (NDUFS1), NADH dehydrogenase ubiquinone 1 beta subcomplex subunit 8, mitochondrial (NDUFB8), Ubiquinol-cytochrome c reductase core protein 2 (UQCR2). In the FoxO signaling pathway (Figure 9), metabolite glutamate was significantly up-regulated, and genes engaged in cell cycle regulation, apoptosis, autophagy and immuno-regulation were affected by *A. veronii* infection (Figure 9). Genes such as FoxO3, cyclin B3 (CCNB3), Cyclin-dependent kinase inhibitor 1B (CDKN1B, p27), Retinoblastoma-like protein 2 (RBL2, p130), Polo-like kinase 2 (PLK2), B-cell lymphoma 6 protein (BCL6) were significantly decreased, and Bcl-2 interacting mediator of cell death (BIM), Interleukin (IL)-6, IL-10, and IL-1β as well as apoptosis-related genes Caspase (CASP) 3, 7 and 8 were increased in group IN (Figure 9a). Genes such as FoxO3, Gamma-aminobutyric acid receptor-associated protein (GABARAP, ATG8), BCL6, cyclin G2 (CCNG2) and CCNB3 were dramatically enhanced in group RE (Figure 9b).

**Figure A4:**
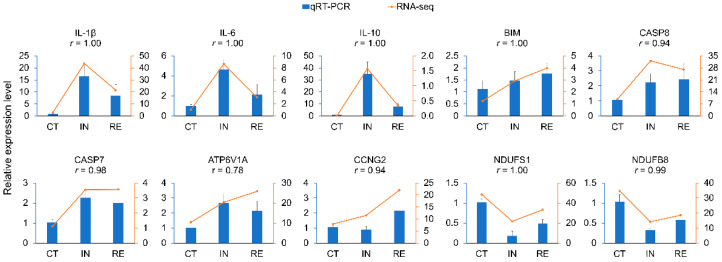
Validation of differentially expressed genes (DEGs) involved in common enriched pathways of the integrated analysis. The expression profiles of ten DEGs were tested by qRT-PCR and compared with that obtained by RNA-seq. The correlation coefficient (*r*) value between the result of qRT-PCR and RNA-seq for each DEG is indicated. For convenience, “CT”, “IN”, and “RE” indicate the control group, *Aeromonas veronii*-infected groups at invaded stage and recovering stage.

To further verify the pathway model, the expressions of ten DEGs (IL-1β, IL-16, IL-10, BIM, CASP7, CASP8, ATP6V1A, CCNG2, NDUFS1 and NDUFB8) involved in the above-mentioned important biological processes were examined by qRT-PCR. The result showed that the expression patterns of these DEGs were consistent with that of RNA-seq, with correlation coefficient (*r*) values ranging from 0.78 to 1.00 (Figure A4).

## 3. Discussion

**Figure 10:**
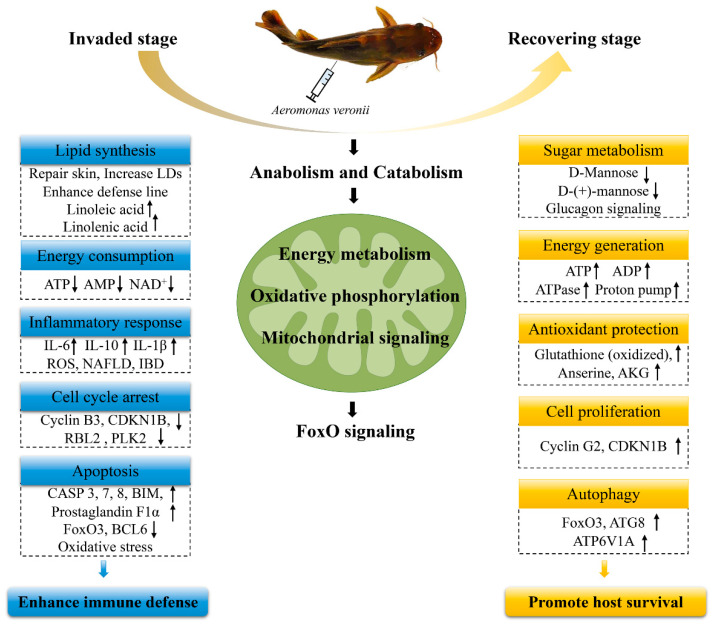
Summary of different defense strategies of yellow catfish in response to *Aeromonas veronii* infection at invaded stage and recovering stage. LDs, lipid droplets. NAFLD, non-alcoholic fatty liver disease. IBD, inflammatory bowel disease. Cyclin B3, G2/mitotic-specific cyclin-B3, also known as CCNB3. CDKN1B, cyclin-dependent kinase inhibitor 1B, also known as p27. RBL2, retinoblastoma-like protein 2, also known as p130. PLK2, serine/threonine-protein kinase PLK2. CASP, caspase. BIM, Bcl-2 interacting mediator of cell death. AKG, α-ketoglutarate, also known as oxoglutaric acid.

In this study, we systematically investigated the comparative transcriptome and metabolome of yellow catfish liver at the invaded stage and recovering stage during *A. veronii* infection. The liver of teleost fish exerts dual effects on metabolism and immune regulation, where interweaves the host defense with metabolic readjustments, representing an ideal material to explore the complex mechanisms underlying the immune defense against pathogen challenge [18]. The integrated analysis of transcriptome and metabolome in our study revealed the high correlation between genes and metabolites, the cooperation of which were effective strategies to cope with the *A. veronii* challenge. Previous studies revealed that variations in gene and/or metabolites expression patterns were responsible for the dying and survival of individuals under stress conditions [10,19]. Similarly, in the present study, the expression profiles of transcripts and metabolites among the three groups were clearly separated, reflecting both DEGs and DAMs were linked to whether the fish were infected or recovered, which were discussed below and summarized in Figure 10.

### 3.1. Anabolism and Catabolism

Body surface ulcers are one of the typical symptoms attributable to *A. veronii* stimulation. At the invaded stage of our study, processes associated with lipid synthesis were initiated through enriched pathways including synthesis and degradation of ketone bodies, primary bile acid biosynthesis, choline metabolism in cancer, glycerophospholipid, linoleic acid, ether lipid, and phospholipase D signaling, as well as the upregulated metabolite lipids such as Fahfa 36:3, Fahfa 36:4, Gamma-linolenic acid, Linoleic acid, Glycerol 3-phosphate and Beta-glycerophosphate. Lipids serve essential roles in structural integrity and functionality in human skin [20], and undoubtedly play critical roles in fish skin, which is the first line of defense against pathogens in fish [21]. Previous studies emphasized the pro-survival role of lipids, for example, unsaturated fatty acid biosynthesis was identified as a pivotal pathway responsible for survival in *E. tarda*-stimulated crucian carps [22]; metabolite linolenic acid and linoleic acid were revealed as biomarkers associated with survival of *S. iniae*-stimulated tilapia [10]. Recently, lipid droplets (LDs), the major lipid storage organelles are proven as an intracellular first line of defense during bacterial infection [23]. Taken together, these results indicate that lipid synthesis at the cost of energy consumption at the invaded stage was likely for skin repairing and LD accumulation, which subsequently strengthened the immune defense line of yellow catfish.

At the recovering stage, nutrition, especially sugar, catabolism (Fat, Lysine and Carbohydrate digestion, as well as Starch and sucrose metabolism), represented the major metabolic processes, which provided sufficient fuels for energy generation. Meanwhile, sugars such as D-Mannose, D-(+)-mannose, Maltotetraose, Maltotriose, and Cellobiose were among the most decreased metabolites, indicating the consumption of sugars exceeded the synthesis at the recovering stage. We also found that Glucagon signaling was activated at this stage, which was likely to maintain glucose homeostasis [24]. It is noteworthy that, investigation on crucian carp proved that decreasing D-Mannose and its involved pathway fructose and mannose metabolism are the most vital biomarkers distinguishing the survivals from deaths during *E. tarda* infection [22]. Together with the observations of the significant decrease of D-Mannose and the involvement of longevity regulating in this study, these results imply sugar metabolism was involved in *A. veronii*-induced response, serving as a survival strategy for yellow catfish.

### 3.2. Energy Metabolism

The TCA cycle and oxidative phosphorylation represent an efficient system to provide energy for entire cells by generating ATP in the mitochondrial inner membrane [25]. In our study, this system was affected by *A. veronii* infection. Specifically, a series of genes in this system, as well as metabolites NAD^+^, AMP and ATP were decreased at the invaded stage, implying an exceeding consumption of energy in host defense against bacterial challenge. A previous study on mice with pathogen stimulation revealed that increasing the proton motive force favored the killing of antibiotic-resistant bacteria, promoting host survival [26]. V-ATPase, also known as H^+^-ATPase, pumps H^+^ into the lumen of vacuolar organelles such as lysosomes [27]. A member of V-ATPase, ATP6V1A, was reported to participate in the defense against salmonella infection of pigs and human autophagy [28,29]. Similarly, in our study, the expression of most key enzymes, especially ATPase, was restored at recovering stage, resulting in dramatically enhancing ATP, which indicates the re-establishing of energy homeostasis, facilitating for survival.

Energy metabolism in mitochondria exerts crucial influences on the host immune response to pathogen infection [30], the intermediates of which serve as signaling regulators controlling immunity [31,32,33]. The mitochondrial signaling elicited by PAMP then triggers inflammatory responses by producing antibacterial ROS and pro-inflammatory cytokines [34,35]. We found that cytokines such as IL-6, IL-10 and IL-1β, were highly enhanced, and meanwhile, pathways of Neomycin, kanamycin and gentamicin biosynthesis and FoxO signaling were implicated at the invaded stage. These results indicated a positive immune defense of yellow catfish against acute bacterial infection. However, excessive inflammatory responses contribute to mitochondrial dysfunction, initiating apoptosis [36,37]. In our study, the functional genes in mitochondria including 20 members of NADH dehydrogenases, two succinate dehydrogenases, two cytochrome c reductases, and seven cytochrome c oxidases were significantly decreased at the invaded stage, reflecting the dysfunction of mitochondria induced by bacterial infection as reported previously [38,39,40]. The mitochondrial dysfunction in turn promotes ROS production and oxidative stress, leading to apoptosis. Accordingly, inflammatory damages such as NAFLD and IBD were found at the invaded stage in our study. Overexpression of UQCRC2 in mice liver was reported to favor the alleviation of liver injury through promoting mitophagy [41]. Suppression on NDUFS1 and NDUFB8 in mammals accompanied by the decline of ATP provoked apoptosis [42,43]. In the present study, UQCRC2, NDUFS1 and NDUFB8 were significantly decreased, meanwhile, Prostaglandin F1α, the pro-apoptosis factor in mammals [44,45], was significantly up-regulated at the invaded stage in the present study, suggesting the engagement of apoptosis in the clearance of the invading pathogen as reported previously in human [46] and fish [47,48]. A study on *Vibrio alginolyticus*-infected zebra fish showed that the dying individuals exhibited excessive levels of inflammatory response compared to survivors [49]. In our study, the antioxidant agents such as AKG [50], Glutathione (oxidized) [51] and Anserine [52], were drastically enhanced at recovering stage, which is likely a protective strategy to avoid exceeding oxidative stress and inflammation. A similar observation was documented in a previous study on piglets, which showed that the supplementation of AKG substantially relieved the LPS-induced liver injury [53].

### 3.3. FoxO Signaling Pathway

FoxO signaling serves vital roles in cellular functions, such as immuno-regulation, apoptosis, autophagy and cell-cycle control [54,55,56], which was widely reported to be implicated in the fish immune response to pathogen infection, including Japanese flounder upon *Vibrio anguillarum* challenge [57,58], largemouth bass upon *Aeromonas hydrophila* challenge [59], and *Carassius auratus gibelio* upon Cyprinid herpesvirus 2 challenge [60]. Glutamate (Glu) serves as an immune-nutrient in fish, provides metabolic fuels for tissues (liver, intestine, and muscle), improves antioxidant capacity, and exerts beneficial effects on immunity [61]. For example, dietary supplementation of Glu and/or glutamine (Gln) promoted serum lysozyme activity and enhanced survival of Nile tilapia [62,63]. In the present study, Glu was among the most increased metabolites, and D-Glutamine and D-glutamate metabolism was among the most enriched pathways after *A. veronii* infection, indicating Glu and Gln as key immune metabolites in the antibacterial defense of yellow catfish.

FoxO3 exerts vital effects on the fish’s innate immune response to pathogens [64]. In LPS-stimulated turbot, diminished FoxO3 significantly promoted the expression of proinflammatory cytokines (such as TNF-α and IL-6), while overexpressed FoxO3 attenuated the LPS-induced hepatic inflammation [65]. In line with this, FoxO3 was found significantly down- and up-regulated at invaded and recovering stages in our study, respectively, which suggests the immune defense via inflammatory response during pathogenic infection, and the protection against excessive inflammation-induced tissue damage during host recovery.

Noteworthily, crosstalk between glutamine and FoxO elucidated that activation of FoxO3 leads to the accumulation of glutamine, which enforced autophagy beneficial for cellular survival; while suppressed FoxO3-mediated autophagy increased apoptosis [55]. Appropriate apoptosis and autophagy are essential survival strategies for anti-microbial defense, which are highly engaged in fish anti-pathogen responses [57,66,67]. Autophagy also favors homeostasis by removing impaired organelles and self-renewal [68]. Genes reported to be associated with the cell cycle in mammals, such as cyclin B [69], CDKN1B [70], RBL2 [71] and PLK2 [72], were inhibited at the invaded stage in this study, indicating the cell cycle arrest in yellow catfish, which was likely the consequence of deficient of energy and inflammatory response. Moreover, we found that CASP 3, 7, 8 and BIM, the apoptosis-related genes in mammals [73], were highly enhanced in this study at the invaded stage, while BCL6, the anti-apoptotic gene in mammals [74], was significantly decreased in yellow catfish at the invaded stage. Combined with the aforementioned decrease of UQCRC2, NDUFS1 and NDUFB8, and increase of Prostaglandin F1α, these observations together highlight that apoptosis activation is likely an important antibacterial strategy of yellow catfish. ATG8 (also known as LC3) represents the marker of autophagy in humans [75], which was significantly up-regulated at recovering stage of *A. veronii*-infected yellow catfish in our study, same as the cell cycle regulatory genes, such as CDKN1B and cyclin G2. These results indicate an elevated activity of autophagy and cellular proliferation, which is likely beneficial for pathogen clearance and host survival.

## 4. Materials and Methods

### 4.1. Sample Preparation

Clinically healthy yellow catfish (~20 g) were obtained from Nanjing Fisheries Research Institute. These fish were acclimated for 2 weeks in the circulating water tanks at 27 ± 1 °C and fed with a commercial diet (Jiaji Feed Co. Ltd., Zhenjiang, China) in the laboratory as reported previously [76]. *A. veronii* was cultured in Luria-Bertani (LB) medium at 28 °C to logarithmic stage, the pellet harvested from which by centrifugation at 2000× *g* for 5 min, was washed and resuspended with PBS as reported previously [57]. Fish in the *A. veronii*-infected group were injected intraperitoneally with 100 μL of the above *A. veronii* suspension (2 × 10^6^ CFU/fish), and fish in the control (CT) group were similarly injected with PBS. At 2 h post-infection (hpi), 6 hpi, 8 hpi, 10 hpi, 12 hpi, 24 hpi, 30 hpi, and 48 hpi, twelve fish were collected in treatment group and control group at each time point. The collected fish were euthanized with MS-222 (Sigma, St. Louis, MO, USA) as described previously [57].

The liver of each fish was taken aseptically, 20 mg of which was used to determine the number of *A. veronii* by plate count as reported previously [77], and the rest were immediately stored in liquid nitrogen for RNA extraction. According to the bacterial loads, samples at 6 hpi were among the increasing trend of bacterial number, and at 12 hpi were among the declined trend, which was termed as *A. veronii* invaded stage (IN) and host recovering stage (RE), respectively. Fish in groups CT, IN and RE were used in this study. At each time point, twelve fish were used in a group. Specifically, every four individuals were pooled as one sequencing sample, and biological triplicates of each group at each time point were applied in the transcriptome. The four individuals from one pool were divided into two metabolomic samples (each sample contained 2 fish), and two biological triplicates were used in metabolome analysis. All infection and collection experimental procedures in this research were in accordance with the Guidelines for the Care and Use of Laboratory Animals in China and were approved by the Nanjing Normal University Animal Ethics Committee (permit No. SYXK2015-0028).

### 4.2. Transcriptome Library Construction and Data Processing

Total RNA was isolated from the liver using Trizol reagent (Invitrogen, CA, USA) following the manufacturer’s instruction. The quality of RNA including concentration, purity and integrity was examined as previously described [57]. The libraries were constructed based on Illumina’s standard protocol for RNAseq library as described in our previous report [57], including total RNA fragmented, first-strand and second-strand cDNA synthesis, and barcoded adapters ligated. The transcriptome libraries were sequenced at the Illumina HiSeq™ 4000 platform.

Data processing was the same as described in our previous study [57]. Briefly, low-quality reads and ribosome RNA (rRNA) were discarded, and the reads mapped to the reference genome of yellow catfish were used for transcript reconstruction for identifying new transcripts. The expression level was normalized to FPKM (Fragments Per Kilobase of transcript per Million mapped reads) [78]. The sequencing data were submitted to the Sequence Read Archive (SRA) with accession no. PRJNA839857.

### 4.3. Differential Expression Analysis and Functional Enrichment Analysis

Differential expression analysis was performed using R package edgeR (v3.12.1) with the exact negative binomial test. Pairwise comparisons were conducted between-group IN and CT, as well as group RE and CT. FDR < 0.05 and log2|(fold change)| > 1 were the criteria for differentially expressed genes (DEGs). The identified DEGs were subjected to KEGG functional analysis based on the KEGG database (http://www.genome.jp/kegg/, accessed on 20 January 2022). A pathway with *p* < 0.05 in the hypergeometric test was considered significantly enriched.

### 4.4. Metabolites Extraction and LC-MS/MS Analysis

The aforementioned liver sample was added into the pre-cold solvent of acetonitrile and methanol (acetonitrile/methanol/water = 2:2:1), followed by vortexed for 30 s, homogenized with a homogenizer, sonicated for 30 min with ice-bath, and incubated at −20 °C for 10 min. The mixture was freeze centrifugated at 14,000× *g* for 20 min. The supernatant was dried in a vacuum centrifuge, and re-dissolved in 100 μL acetonitrile solvent (acetonitrile /water = 1:1) for LC-MS/MS analysis. To examine the stability and repeatability, QC samples were prepared by pooling an equal aliquot of each sample and analyzed together with experimental samples. LC-MS/MS analysis was performed using a UHPLC (1290 Infinity LC, Agilent Technologies) coupled to a quadrupole time-of-flight (AB Sciex TripleTOF 6600) in Shanghai Applied Protein Technology Co., Ltd., according to the manufacturer’s instruction.

### 4.5. Metabolome Data Processing and Multivariate Statistical Analysis

In order to enhance the detection coverage, both POS and NEG were used to identify metabolites. PCA was carried out with experimental samples and QC samples for quality control using R language gmodels (v2.18.1) (https://CRAN.R-project.org/package=gmodels, accessed on 20 January 2022). The abundance of metabolite was normalized by z-score and subjected to hierarchical clustered heatmap analysis using R package pheatmap to exhibit the relationship and expression patterns of metabolites in all samples. Multivariate statistical analysis was performed by OPLS-DA (orthogonal projection to latent structures-discriminant analysis) using R package models (http://www.r-project.org/, accessed on 24 January 2022) Cross-validation and permutation test [79] was applied to further verify the reliability of OPLS-DA model.

### 4.6. DAMs Identification and KEGG Enrichment Analysis

The VIP score of the OPLS-DA model and T-test were used to rank the metabolites that best distinguished between the treatment group and control group. A metabolite with VIP > 1 and *p* < 0.05 was considered as a DAM. The z-score of DAMs was calculated to measure the relative content of metabolites at the same level. KEGG enrichment analysis was applied for DAMs based on the KEGG database (http://www.genome.jp/kegg/, accessed on 24 January 2022).

### 4.7. Integrated Analysis of Transcriptomic and Metabolomic Data

Two models were used for integrated analysis in this study. The O2PLS (two-way orthogonal PLS) model was applied to analyze the integrative data of transcriptome and metabolome for modeling systematic variation using the R package OmicsPLS [80]. O2PLS decomposes the total variation of two omics data matrices X and Y and assesses the contribution of variation explanation with R2. The join loading plots reflecting the general correlation strength between genes and metabolites were constructed based on the loading values in O2PLS. The pathway model was used to explore the links of genes and metabolites in biological pathways according to the commonly involved KEGG pathways and visualized by Pathview [81,82].

### 4.8. Experimental Validation of DEGs

The expression patterns of ten DEGs engaged in the important pathways were tested by qRT-PCR. Specific primers for each DEG were designed using Primer designer of NCBI (https://www.ncbi.nlm.nih.gov, accessed on 18 May 2022) and the sequences were summarized in Table A1. qRT-PCR reactions were performed in the ABI Step One Plus system (Applied Biosystems, Foster, CA, USA) using SYBR Green Master mix (Hieff UNICON, Shanghai, China) following the manufacturer’s protocol. The expression level of each DEG was calculated using 2^−ΔΔCt^ comparative Ct method with β-actin as an internal control. Correlation analysis between the results obtained from qRT-PCR and RNA-seq was conducted using cor.test in R (v3.5.2) (https://cran.r-project.org, accessed on 1 July 2019) [83] as reported previously [57].

## 5. Conclusions

This study provides the first integrated and comparative analyses of the transcriptome and metabolome of yellow catfish in response to *A. veronii* infection at the invaded stage and recovering stage. The key biomarkers, important pathways and specific responses were determined. We found that at the *A. veronii* invading stage, the immune defense of yellow catfish was potentiated by synthesizing lipid costing ATP to maintain skin barrier integrity, and intracellular defense line via LD accumulation, triggering inflammatory response with mitochondrial signaling and activating apoptosis accompanied by cell cycle arrest (Figure 10). At the fish recovering stage, survival strategies including sugar metabolism, energy generation, antioxidant protection, cell proliferation and autophagy were highlighted (Figure 10). Our results provide valuable datasets for future delving into the immune mechanisms of yellow catfish and add new insights into the antibacterial defense of fish.

## Figures and Tables

**Table 1:** Summary of data processing of transcriptome libraries.

Sample	Raw Reads	HQ Reads	HQ Ratio (%)	RHQ Reads	Mapped Reads	Mapping Ratio (%)
CT-a	78,771,364	78,499,694	99.66	45,734,330	43,105,538	94.25
CT-b	76,322,050	76,044,322	99.64	40,809,162	38,653,690	94.72
CT-c	79,193,264	78,939,460	99.68	57,590,950	54,559,150	94.74
IN-a	89,965,810	89,748,254	99.76	66,765,870	62,919,273	94.24
IN-b	99,306,580	99,037,870	99.73	73,854,352	69,466,476	94.06
IN-c	91,103,442	90,859,558	99.73	52,051,328	48,980,490	94.10
RE-a	80,482,820	80,287,406	99.76	51,564,106	49,152,222	95.32
RE-b	85,969,140	85,755,306	99.75	52,182,432	49,328,966	94.53
RE-c	76,547,964	76,350,666	99.74	47,252,734	44,118,118	93.37

For convenience, “CT”, “IN”, and “RE” indicate the control group, *Aeromonas veronii*-infected groups at invaded stage and recovering stage. “a”, “b”, and “c” indicate three biological triplicates. HQ, high quality. RHQ, the remaining high quality.

## Data Availability

Not applicable.

## Supplementary Materials

The supporting information can be downloaded at: https://www.mdpi.com/article/10.3390/ijms231710121/s1.

## Appendix A

**Table A1:** Summary of primers used for qRT-PCR validation.

Name	Forward Primer (5′ to 3′)	Reverse Primer (5′ to 3′)
IL-1β	AGCCTGTGTGTTTGGGGATTGTG	CGCTCCATTCCATCGTTCTCCTTG
IL-6	ACACTCCATTCTGCACAGCTTCAC	CCACTTGTCCACCTTCTTGTCCTC
IL-10	TACTTGGAGACCGTGTTGCC	GCCTTTTCCTCCCATTCGAC
BIM	GCTCCTGATTGGTCGGCTGTTAC	CAGTCTGGTGCTACTTGTCTGCTG
CASP8	TTCTGCCCGAGTCTCATCAATAAGC	TCTCCTCTCCTGCATTCTCTTCCAC
CASP7	TTGCGAGACTCTGAGGAGGA	CCTTGTTCTTCTTGCCTCCA
ATP6V1A	CCAAGAGTTTACGGGTCGGG	TCCCTCGGTTTTTAGGTGGC
CCNG2	CTGCTGGAGTGAGAGTGAGGA	AGGGAAATAAGGCCCATCTGC
NDUFS1	TCATCGCGCCGGATATTGAA	AACGCAACATGACGCTTGAC
NDUFB8	CCGTATCCTCGCACTGCTGAAG	GTAATCGCCATAGCCCTGTCCATC
β-actin	TTCGCTTGGAGATGATGCT	CGTGCTCAATGGGGTACT
